# Comparison of neoadjuvant and adjuvant chemotherapy for operable triple-negative breast cancer before the era of immune checkpoint inhibitors: A retrospective study from the Japanese National Clinical Database-Breast Cancer Registry

**DOI:** 10.1016/j.breast.2025.104460

**Published:** 2025-03-25

**Authors:** Tomoe Taji, Hiraku Kumamaru, Yuki Kataoka, Kotaro Iijima, Hirofumi Suwa, Hiroshi Ishiguro, Naruto Taira, Takanori Ishida, Shigehira Saji

**Affiliations:** a Department of Breast Surgery, Kansai Medical University Hospital, 3-1 Shinmachi 2 Chome, Hirakata, Osaka, 573-1191, Japan; b Department of Breast Surgery, Hyogo Prefectural Amagasaki General Medical Center, 2-17-77, Higashinaniwa-cho, Amagasaki, Hyogo, 660-8550, Japan; c Department of Healthcare Quality Assessment, Graduate School of Medicine, The University of Tokyo, 7-3-1, Hongo, Bunkyo-ward, Tokyo, 113-8655, Japan; d Department of Internal Medicine, Kyoto Min-iren Asukai Hospital, 89, Tanakaasukai-cho, Sakyo-ku, Kyoto, 606-8226, Japan; e Scientific Research Works Peer Support Group (SRWS-PSG), Osaka, Japan; f Department of Healthcare Epidemiology, Kyoto University Graduate School of Medicine/School of Public Health, Yoshida Konoe-cho, Sakyo-ku, Kyoto, 606-8303, Japan; g Department of International and Community Oral Health, Tohoku University Graduate School of Dentistry, 4-1, Seiryo-machi, Aoba-ku, Sendai, Miyagi, 980-8575, Japan; h Breast Oncology Center, Cancer Institute Hospital, Japanese Foundation for Cancer Research, 3-8-31, Ariake, Koto-ku, Tokyo, 135-8550, Japan; i Breast Oncology Service, Saitama Medical University International Medical Center, 1397-1, Yamane, Hidaka, Saitama, 350-1298, Japan; j Department of Breast and Thyroid Surgery, Kawasaki Medical School, 577 Matsushima, Kurashiki City, Okayama, 701-0192, Japan; k Department of Breast and Endocrine Surgical Oncology, Tohoku University Graduate School of Medicine, Sendai, 980-8575, Japan; l Department of Medical Oncology, Fukushima Medical University, 1, Hikariga-oka, Fukushima, Fukushima, 960-1295, Japan

**Keywords:** Triple-negative, Breast cancer, Neoadjuvant chemotherapy

## Abstract

**Background:**

While neoadjuvant chemotherapy (NAC) is recommended for stage II-III triple-negative breast cancer (TNBC), its equivalence to adjuvant chemotherapy (AdjC) has been questioned based on a retrospective study using the National Cancer Database in the United States, which lacked adjustment for important covariates. Given the unlikelihood of new randomized trials being conducted, well-designed, large-scale, retrospective studies are needed.

**Patients and methods:**

We retrospectively analyzed operable TNBC patients from the Japanese National Clinical Database- Breast Cancer Registry (2012–2016). Inclusion criteria were clinical stage I-IIIB, estrogen receptor (ER) < 10 %, progesterone receptor (PgR) < 10 %, and HER2-negative. We excluded patients with carcinoma in situ, cT4a/T4c/T4d, cN3, cM1, bilateral breast cancer, male, non-epithelial tumor, no chemotherapy, no surgery and no follow-up. Primary and secondary outcomes of overall survival (OS) and recurrence-free survival (RFS) were compared between NAC and AdjC using Cox proportional Hazard regression among the exact matched cohort based on age, BMI, cT, cN, histology, ER/PgR positivity, chemotherapy regimen, breast operative technique, radiotherapy, and institution size.

**Results:**

Among 9,000 AdjC and 5,520 NAC patients, 3,256 matched cases were compared. OS and RFS were significantly worse for patients with NAC (Hazard Ratio 1.45 (95 % confidence interval 1.26–1.68) and 1.33 (1.19–1.49), respectively), particularly in patients <65 years, with stage II-IIIB, and with invasive ductal carcinoma.

**Conclusion:**

Patients with NAC had worse prognosis, possibly due to unadjusted confounders. Although the availability of immune checkpoint inhibitors (ICIs) limits the clinical impact, the result could provide supplemental insights for treatment decisions in patients who are not candidates for ICIs.

## Highlights

• We retrospectively analyzed operable triple-negative breast cancer patients from the Japanese National Clinical Database to compare the prognosis between neoadjuvant chemotherapy (NAC) and adjuvant chemotherapy.
• After exact matching, 3,256 each were compared.
• Overall survival and recurrence-free survival were significantly worse for patients with NAC (HR 1.45 (95 %CI 1.26–1.68) and 1.33 (1.19–1.49), respectively).
• Although the availability of immune checkpoint inhibitors (ICIs) limits the clinical impact, the result could provide supplemental insights for treatment decisions in patients who are not candidates for ICIs.

## 1 Introduction

Breast cancer is classified into intrinsic subtypes by gene expression patterns of cDNA microarrays [1]. Basal-like is a well-known intrinsic subtype with a poor prognosis. In clinical practice, the intrinsic subtype is substituted by a subtype based on immunohistochemical staining. Basal-like is considered generally consistent with triple-negative (ER-negative, PgR-negative, and HER2-negative). Current guidelines recommend neoadjuvant chemotherapy (NAC) as the standard treatment for patients with stage II and III triple-negative breast cancer (TNBC) [2].

Researchers have extensively studied the comparative efficacy of NAC and adjuvant chemotherapy (AdjC) in operable breast cancer. A meta-analysis by the Early Breast Cancer Trialists' Collaborative Group (EBCTCG) demonstrated comparable outcomes for distant recurrence and breast cancer mortality between NAC and AdjC in operative breast cancer patients [3]. However, recent studies have raised questions about this equivalence in TNBC patients [[4], [5], [6], [7], [8], [9]]. A retrospective study using the National Cancer Database in the United States reported worse overall survival in the NAC group for stage II and III TNBC patients [7]. Nevertheless, this study lacked adjustment for important covariates such as age, stage, and breast surgery technique. Moreover, the EBCTCG meta-analysis included outdated trials, predominantly using anthracycline alone, and lacked HER2 information.

Given the limited likelihood of new randomized trials comparing NAC and AdjC in operable TNBC being conducted, we carried out a retrospective study using the Japanese National Clinical Database- Breast Cancer Registry. This database offers data on both overall survival (OS) and recurrence-free survival (RFS), as well as prognostic covariates. We hypothesized that after adjusting for the confounding factors, the prognosis for NAC and AdjC will be equivalent in operable TNBC patients. Our study aims to compare the outcomes of NAC and AdjC in patients with operable TNBC using this comprehensive Japanese database.

## 2 Patients and methods

### 2.1 Data source

We used the Japanese National Clinical Database- Breast Cancer Registry (NCD-JBCR). This database is linked to a board certification system of the Japan Surgical Society as well as the Japanese Breast Cancer Society. As of 2016, the NCD has 656,896 breast cancer patients’ data registered from more than 1,400 facilities, covering more than 80 % of breast cancer cases in Japan [10]. Patient characteristics, clinicopathological factors, recurrence and survival data were registered through a web-based system. Histological classification was performed according to the General Rules for Clinical and Pathological Recording of Breast Cancer [11] and further assessed according to the Classification of Tumors of the Breast and Female Genital Organs [12].

We followed the RECORD statement to report this study (Table S1).

### 2.2 Patients

**Fig. 1:**
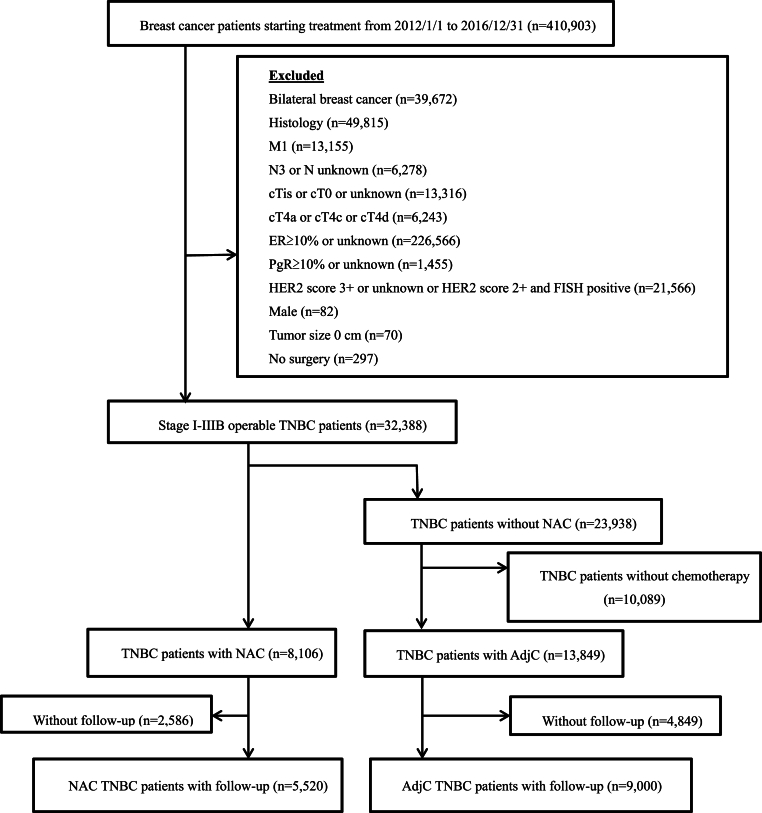
Patient flow diagram.

We enrolled breast cancer patients diagnosed and treated from January 1st^,^ 2012 to December 31st^,^ 2016. This secured a minimum follow-up period of 5 years + 1 year for registration for all patients at the time of data analysis. Our inclusion criteria were operable triple negative breast cancer (TNBC), defined as clinical stage I-IIIB (excluding cT4a, cT4c and cT4d), ER<10 %, PgR<10 %, and HER2-negative. We excluded patients with carcinoma in situ, cT4a, cT4c, cT4d, cN3, cM1, bilateral breast cancer, male sex, non-epithelial tumor, no chemotherapy, and no surgery. We also excluded those with no follow-up data registration, those with follow-up of 30 days or less, and those with erroneous registration of time to recurrence (Fig. 1). We did not exclude the patients who received both NAC and AdjC. Excluding these patients may skew the result in favor of NAC.

### 2.3 Exposures

Patients who received chemotherapy preoperatively and then underwent surgery were defined as the NAC group, while those who underwent surgery and then received postoperative chemotherapy were defined as the AdjC group. Chemotherapy regimens were not restricted. Cytotoxic agents other than anthracyclines and taxanes, which are standard in the perioperative chemotherapy of breast cancer, were allowed. Patients receiving chemotherapy both preoperatively and postoperatively are included in the NAC group. Anthracycline was defined as chemotherapy including AC (doxorubicin and cyclophosphamide), EC (epirubicin and cyclophosphamide), CAF (fluorouracil, doxorubicin and cyclophosphamide), CEF (fluorouracil, epirubicin and cyclophosphamide), ET (epirubicin and docetaxel) and epirubicin. Taxane was defined as chemotherapy including ET, DTX (docetaxel), PTX (paclitaxel), nab-PTX and TC (docetaxel and cyclophosphamide). Others were defined as regimens that did not include either anthracycline or taxane.

### 2.4 Outcome measures

The primary outcome was OS. Secondary outcome was RFS. OS was defined as the time from the earlier date of the breast cancer surgery or initiation of chemotherapy to all-cause death. RFS was defined as the time from the earlier date of the breast cancer surgery or initiation of chemotherapy to recurrence or death, whichever occurs first.

### 2.5 Cofounding factors

Fig. S2 presents a directed acyclic graph (DAG) depicting causal relationships between factors influencing neoadjuvant chemotherapy decision-making and subsequent breast cancer relapse and mortality outcomes. The DAG enables the identification of confounding factors requiring statistical adjustment, intermediate variables in causal pathways, and factors excluded from adjustment in the current analysis. This structured visualization supports our methodological approach to addressing potential biases in treatment effect estimation. The complete methodological rationale for variable selection based on this causal framework appears on page 8. We considered age, BMI, cT, cN, histologic type (adenoid cystic carcinoma and medullary carcinoma have a better prognosis than invasive ductal carcinoma), and hormone receptor status as cofounding factors (Fig. S1). We considered chemotherapy regimen, breast surgery technique, and radiation therapy as intermediate factors.

Nuclear grade (NG) at biopsy was not collected and postoperative NG was available only from 2013 onwards. This was not considered as a confounding factor as in the NAC group, postoperative NG is affected by therapeutic effect. Although positive margins have been reported to increase local recurrence, we did not add this as a covariate as the pathological evaluation of margins is not standardized among hospitals and were only available in those registered after 2014. Based on our preliminary assessment in the yearly change in the use of NAC during the study period (Fig. S2), we did not consider calendar time as a confounder.

### 2.6 Comparing pCR vs. non-pCR

Previous studies have shown that patients with pathological complete response (pCR) have a better prognosis than those without pCR. We evaluated the OS and the RFS between pCR and non-pCR in the NAC group. Since no data about therapeutic effects or pathological response were collected in the database during the study period, we defined pCR as the invasive tumor diameter of 0 cm or unknown size, and no lymph node metastasis.

### 2.7 Statistical analysis

We performed descriptive statistics using summary statistics. Survival curves were estimated using the Kaplan-Meier method, and comparisons between the two groups were performed using the log-rank test. HRs and confidence intervals for OS and RFS were calculated using the Cox proportional hazards model.

Patients in the NAC group were matched at 1:1 ratio to the patients in the AdjC group based on the following ten covariates: age (−65, 66–70, 71-), cT (T1, T2-4b), cN (N0, N1-2), histology (IDC, medullary or adnoid cystic carcinoma, others), BMI (−25, 25<), chemotherapy regimen (A (anthracycline)-T (taxane), A, T, others), breast surgery technique (Bt (total mastectomy), Bp (partial mastectomy), others), radiation therapy, hormone receptor status (ER 0 % and PgR 0 %, others), and whether the patient was treated in a high-volume facility (annual breast cancer surgeries per facilities <100, 100-). As subgroup analyses, we compared OS and RFS between NAC and AdjC in groups based on age, ER/PgR positivity, histological type and cStage.

The five-year survival among patients with stages II and III disease were compared with the results of the retrospective study using National Cancer Database in the United States [7].

Statistical analysis was performed using SAS version 9.4. A two-sided test was adopted, with p < 0.05 indicating statistical significance.

### 2.8 Ethical considerations

The study protocol was approved by the Ethics Committee of Hyogo Prefectural Amagasaki General Medical Center (approval number 4–48). The need for patient consent was waived because the patient records were anonymized and de-identified before analysis.

## 3 Results

After applying the exclusion criteria, we included 14,520 TNBC patients who had undergone surgery and chemotherapy and were followed up after 5 years. We finally analyzed 5,520 patients in the NAC group and 9,000 patients in the AdjC group (Fig. 1). The median follow-up time was 1,825 days, with a range of 31 to 1,825 days. The inclusion of patients with shorter follow-up periods enabled the capture of early recurrence events, which represent a hallmark characteristic of TNBC biology. Analysis of these cases provides valuable insights into the natural course of disease progression, particularly regarding locally detectable recurrences that emerge before scheduled surveillance imaging.

Table 1 shows that TNBC patients who received NAC were younger (<66 years old in 81 % vs. 66 %), had more invasive ductal carcinoma (91 % vs. 83 %), had less cT1 (21 % vs. 48 %), had less cN0 (48 % vs. 79 %) and were treated in high volume centers (70 % vs. 59 %). In terms of treatment, Table 2 indicates more patients with NAC received anthracycline plus taxane regimens (78 % vs. 42 %) and radiation therapy (63 % vs. 43 %). Nuclear grade 3 was less common in the NAC group (30 % vs. 55 %). The possible reason was therapeutic effects. Unexpectedly positive margins were not increased in the NAC group. Thirteen patients in NAC and four patients in AdjC received carboplatin which was not covered by Japanese national insurance. No patients in either group were administered only carboplatin without anthracycline and taxane (Table S2).

**Table 1:** Baseline characteristics.

	AdjC		NAC	
	N = 9000	(%)	N = 5520	(%)
Age				
−65	5891	65.5	4481	81.2
66–70	1366	15.2	625	11.3
71-	1743	19.4	414	7.5
Menopausal status				
Premenopause	2339	26.0	2257	40.9
Postmenopause	6404	71.2	3111	56.4
Unknown	257	2.9	152	2.8
BMI (kg/m^2^)				
−25.0	7016	78.0	4200	76.1
25.0<	1984	22.0	1320	23.9
Histology				
IDC	7438	82.6	5016	90.9
Medullary or adenoid cystic carcinoma	261	2.9	72	1.3
Others	1301	14.5	432	7.9
Tumor diameter				
0.1–2.0 cm	3665	40.7	1030	18.7
2.1–5.0 cm	4653	51.7	3620	65.6
5.1 cm-	499	5.5	800	14.5
Unknown	183	2.0	70	1.3
Clinical T status				
T1	4349	48.3	1159	21.0
T2	4174	46.4	3445	62.4
T3	294	3.3	475	8.6
T4b	183	2.0	441	8.0
Clinical N status				
N0	7118	79.1	2628	47.6
N1	1678	18.6	2402	43.5
N2	204	2.3	490	8.9
ER				
Negative	7972	88.6	4,840	87.7
1–9 %	654	11.4	680	12.3
PgR				
Negative	8,346	92.7	5,155	93.4
1–9 %	654	7.3	365	6.6
HER2				
Score 0	5011	55.7	3031	54.9
Score 1	2867	31.9	1830	33.2
Score 2 and FISH negative	1122	12.5	659	11.9
Annual breast cancer cases per facility				
−99	3678	40.9	1655	30.0
100-	5322	59.1	3865	70.0

Abbreviations: BMI = body mass index; IDC = invasive ductal carcinoma; ER = estrogen receptor; PgR = progesterone receptor; HER2 = human epidermal growth receptor 2; FISH = Fluorescence in situ hybridization.

**Table 2:** Postoperative pathological characteristics and treatment.

	AdjC		NAC	
	N = 9000	(%)	N = 5520	(%)
Invasive diameter				
0 cm	6	0.1	1020	18.5
0.1–2.0 cm	4591	51.0	2253	40.8
2.1–5.0 cm	3757	41.7	1081	19.6
5.1 cm-	560	6.2	388	7.0
Missing	86	1.0	778	14.1
Node metastases				
None	6123	68.0	3869	70.1
1–3 nodes	1887	21.0	947	17.2
4–9 nodes	489	5.4	298	5.4
10 nodes or more	289	3.2	139	2.5
Missing	212	2.4	267	4.8
NG (postoperative) (y = 2013–2016)				
NG 1	594	6.6	496	9.0
NG 2	1541	17.1	895	16.2
NG 3	4520	50.2	1722	31.2
Not tested or unknown	447	5.0	1808	32.8
Missing	1898	21.1	599	10.9
Margin (y = 2014–2016)				
Negative	4855	53.9	3433	62.2
Positive	235	2.6	295	5.3
Unknown	200	2.2	102	1.8
Missing	3710	41.2	1690	30.6
Breast surgery				
Bt	4473	49.7	2596	47.0
Bp	4379	48.7	2825	51.2
Others	148	1.6	99	1.8
Axillary surgery				
None	215	2.4	231	4.2
SN	5399	60.0	2,129	38.6
Ax	3,197	35.5	3,013	54.6
Sampling	170	1.9	86	1.6
Others or unknown	19	0.2	61	1.1
Chemotherapy				
Anthracyclin-taxane	3794	42.2	4284	77.6
Anthracycline	2057	22.9	755	13.7
Taxane	2138	23.8	394	7.1
Others	1011	11.2	93	1.7
Endocrine therapy	682	7.6	591	10.7
Radiotherapy	3867	43.0	3457	62.6

NG = nuclear grade; Bt = total mastectomy; Bp = partial mastectomy; Ax = axillary lymph node dissection; SN = sentinel node biopsy.

After exact matching for age, BMI, cT, cN, histology, ER/PgR positivity, chemotherapy regimen, breast operative technique, radiation therapy, and institution size, 3256 cases each were compared (Table 3).

**Table 3:** Patient characteristics and treatment after exact matching.

	AdjC		NAC	
	N = 3256	(%)	N = 3256	(%)
Age				
−65	2580	79.2	2580	79.2
66-70	399	12.3	399	12.3
71-	277	8.5	277	8.5
BMI >25.0	723	22.2	723	22.2
cT2-	2281	70.1	2281	70.1
cN1-	1165	35.8	1165	35.8
Histology				
IDC	2946	90.5	2946	90.5
Medullary or adenoid cystic carcinoma	32	1.0	32	1.0
Others	278	8.5	278	8.5
ER 0 % and PgR 0 %	2835	87.1	2835	87.1
Chemotherapy				
Anthracyclin-taxane	2311	71.0	2311	71.0
Anthracyclin	553	17.0	553	17.0
Taxane	328	10.1	328	10.1
Others	64	2.0	64	2.0
Breast surgery				
Bt	1653	50.8	1653	50.8
Bp	1583	48.6	1583	48.6
Other	20	0.6	20	0.6
Radiotherapy	1688	51.8	1688	51.8
Annual breast cancer surgeries per facilities <100	1108	34.0	1108	34.0
Invasive diameter				
0 cm	1	0.0	601	18.5
0.1–2.0 cm	1261	38.7	1369	42.0
2.1–5.0 cm	1724	52.9	639	19.6
5.1 cm-	251	7.7	194	6.0
Missing	19	0.6	453	13.9
Node metastases				
None	1843	56.6	2455	75.4
1–3 nodes	913	28.0	460	14.1
4–9 nodes	288	8.8	121	3.7
10 nodes or more	174	5.3	67	2.1
Missing	38	1.2	153	4.7
NG (postoperative) (y = 2013–2016)				
NG 1	152	4.7	314	9.6
NG 2	502	15.4	546	16.8
NG 3	1788	54.9	970	29.8
Not tested or unknown	129	4.0	1098	33.7
Missing	685	21.0	328	10.1
Margin (y = 2014–2016)				
Negative	1734	53.3	2057	63.2
Positive	92	2.8	50	1.5
Unknown	68	2.1	175	5.4
Missing	1362	41.8	974	29.9
Axillary surgery				
None	40	1.2	157	4.8
SN	1596	49.0	1603	49.2
Ax	1563	48.0	1431	43.9
Sampling	52	1.8	51	1.6
Others or unknown	5	0.1	14	0.4

**Fig. 2:**
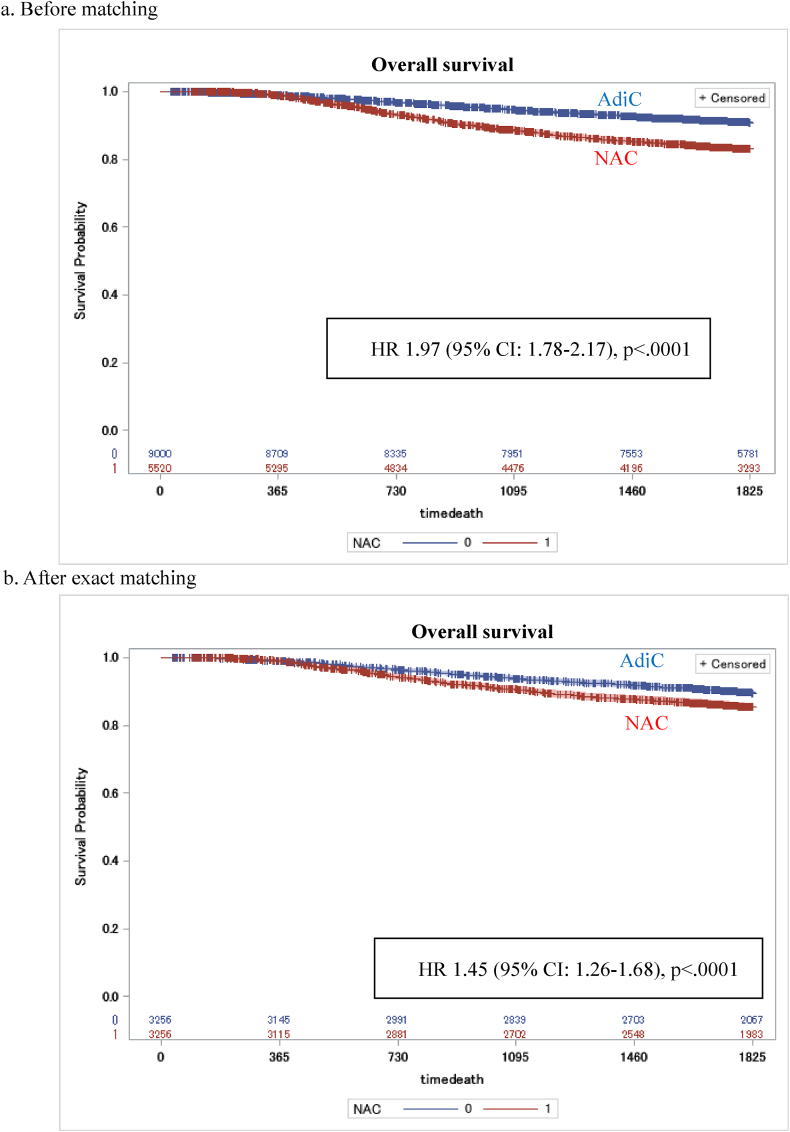
Kaplan-Meier curves comparing OS between TNBC patients with NAC and those with AdjC.

**Fig. 3:**
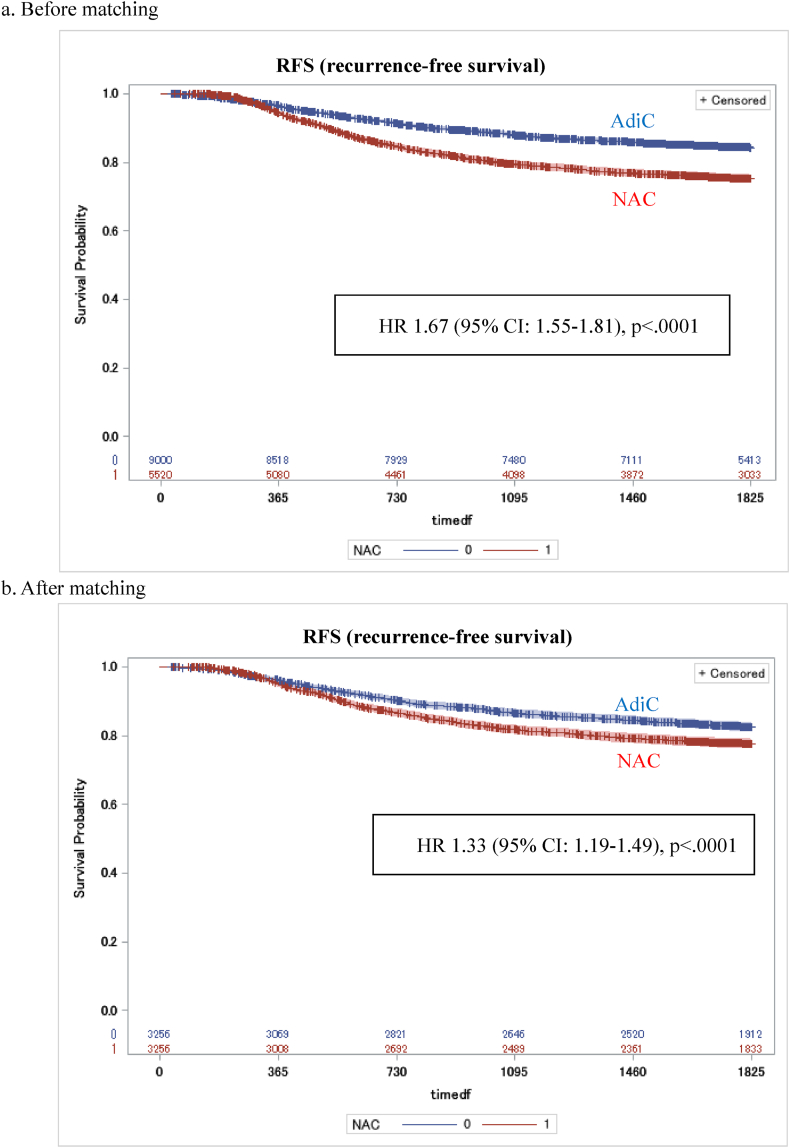
Kaplan-Meier curves comparing RFS between TNBC patients with NAC and those with AdjC.

**Fig. 4:**
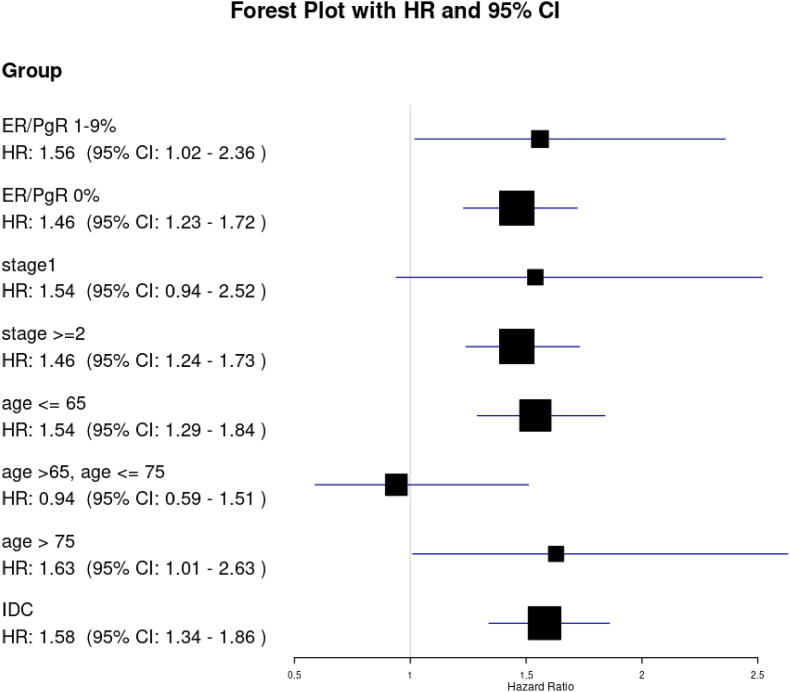
Exploratory analysis of OS in Subgroups.

**Fig. 5:**
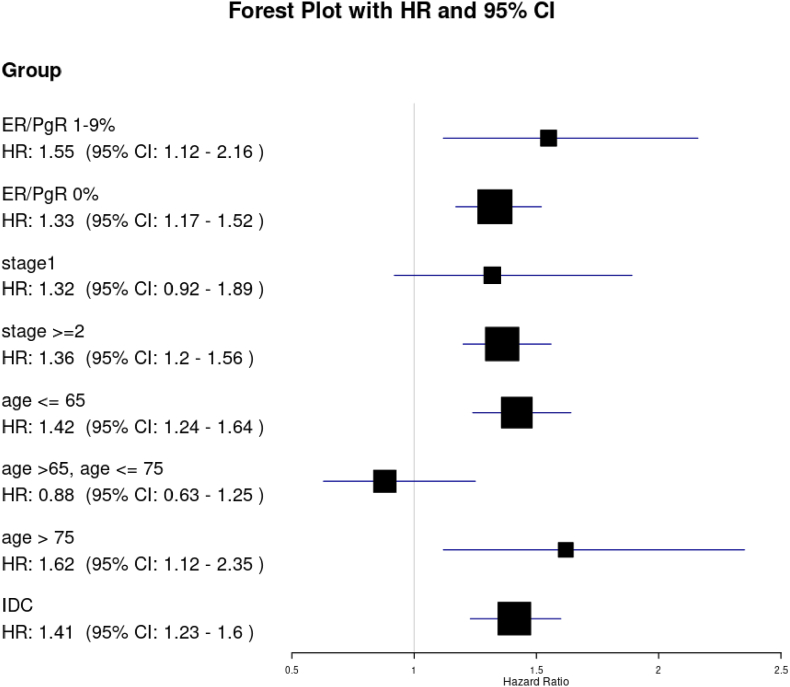
Exploratory analysis of RFS in subgroups.

Both overall survival (HR 1.45; 95 % CI, 1.26–1.68, p < .0001) and recurrence-free survival (HR 1.33; 95 % CI, 1.19–1.49, p < .0001) were worse for patients with NAC than those with AdjC (Fig. 2, Fig. 3). In subgroup analysis, TNBC patients who receive NAC had worse OS and RFS in those younger than 65 years, in stage II or higher, and with invasive ductal carcinoma (Fig. 4, Fig. 5).

The NAC group had more local recurrences and distant metastases as well as breast cancer deaths (Table 4). Death from other diseases was more frequent in the NAC group (39 patients vs. 26 patients).

**Table 4:** First invasive disease-free survival event after exact matching.

	Before matching		After matching	
	AdjC (N = 9000)	NAC (N = 5520)	AdjC (N = 3256)	NAC (N = 3256)
First recurrence site				
Distant	903	940	407	497
Lung	374	444	164	243
Bone	295	262	127	141
Liver	181	251	85	138
Brain	121	176	64	97
Distant node	192	244	98	114
Pleura	80	82	46	41
Others	113	107	54	66
Locoregional	682	626	266	340
Ipsilateral breast	159	137	60	70
Ipsilateral axillary node	320	214	112	121
Others	381	431	167	230
Death	766	868	316	440
Breast cancer	604	777	277	389
Other cause	119	66	26	39
Unknown cause	43	25	13	12

The pCR rate was 26.9 %. Both the OS and the RFS were significantly better in the pCR group (Fig. S3).

Among the stage II and III patients, 5-year OS rates for both the NAC and AdjC groups were higher in our study (83.0 % vs 87.9 %, respectively) than in the retrospective study using the National Cancer Database in the United States (Fig. S4) [7] (73.4 % vs 76.8 %, respectively).

## 4 Discussion

NAC was associated with poorer OS and RFS compared with AdjC after adjustments for the important confounders including age, BMI, cT, cN, histologic type and hormone receptor status. This result was consistent across subgroups but seemed robust in those younger than 65, with stage II or higher, and with invasive ductal carcinoma.

Despite the equivalence of outcomes between NAC and AdjC shown in the meta-analysis [3], this study reported the worse OS in the TNBC patients receiving NAC, as in the retrospective study using the National Cancer Database in the United State [7]. Nevertheless, the previous study had no recurrence data and lacked adjustment for important covariates such as age, stage, and breast surgery technique.

Possible reasons for worse OS in the NAC group include the following. First, progressive disease during NAC could cause early relapse and breast-cancer related death. Second, adverse events due to chemotherapy could delay NAC or surgery. Third, some patients might experience treatment-related death. Forth, unmeasured confounding factors such as preoperative nuclear or histological grade, Ki-67, comorbidities, and time from diagnosis to start of treatment (chemotherapy in the NAC group and surgery in the AdjC group) were not adjusted due to the retrospective design. Fifth, The NCD lacked information on treatment outcomes by NAC and details necessary for calculating relative dose intensity. Sixth, the possibility remained that the risk of recurrence was higher in the NAC group even with the exact matching of cT and cN. NAC is indicated when the tumor size is too large for breast-conserving surgery, or when postoperative chemotherapy is needed, according to the Japanese Breast Cancer Society Clinical Practice Guideline for systemic treatment of breast cancer [13]. It is difficult to accurately measure tumor diameter and count the number of lymph node metastases before surgery. Moreover, we have no information on the method to measure tumor diameter (whether palpation, mammography, ultrasonography, or MRI was used), and the method to diagnose lymph node metastases (whether cytology, histology, or imaging was mandatory).

The strengths of this study are that the Japanese NCD-BCR included a large number of TNBC patients, information on relapse as well as survival, and details of pathological findings and treatment.

There were several limitations. First, nuclear or histological grades and Ki-67 of biopsy specimens were not collected in the NCD at the time of the study. Before 2017, NAC was not the standard of care in stage II or III TNBC patients until the St. Gallen guidelines were published [14]. Therefore, patients with NAC might have had higher grade or Ki-67 and resulted in more recurrence. Second, we excluded patients who were no longer operable after NAC because their tumors have increased or when distant metastases have appeared during NAC. This exclusion may have skewed the results in favor of NAC. Third, relative dose intensity (RDI) was not available. In NAC group, we use the time from the start of chemotherapy to surgery as a surrogate for RDI. Standard treatment was considered 12 weeks for anthracycline or taxane only and 24 weeks for anthracycline and taxane. We set the optimal treatment periods were 84–105 days and 126–210 days, respectively, to maintain 80 % RDI (Table S3). While 77.8 % had anthracycline and taxane in the optimal periods, only 28.2 % received anthracycline or taxane only. Lower RDI may have resulted in the worse outcome in the NAC group. We could not compare the treatment periods between NAC and AdjC because we did not have the data about treatment duration in the AdjC group. Fourth, we did not include data about comorbidities such as coronary artery disease, cerebrovascular disease, malignancies other than breast cancer, diabetes, heart failure, and COPD, since they were only available after 2016. Patients with severe comorbidities are more likely to opt for prior surgery, which may work in favor of NAC. Fifth, the start of survival was not the date of breast cancer diagnosis which was not available in NCD-BCR. In other words, the time from diagnosis to surgery in the AdjC group and the time from diagnosis to the start of preoperative chemotherapy in the NAC group were not considered. This may have worked in favor of the NAC group. Time interval from breast cancer diagnosis to definitive surgery is increasing [15] and several literatures reported that treatment delay is associated with worse survival [16,17]. Sixth, the pathological complete response rate of 26.9 % observed in the current study was lower than the rate reported in recent clinical trials such as KEYNOTE-522 (control arm: 51.2 %) [^18,19^]. Three factors may explain this difference. First, chemotherapy intensity remained suboptimal in many cases with shorter or longer time periods from the start of NAC to surgery (Table S3). Second, only 77.6 % of patients who received neoadjuvant chemotherapy were administered the recommended anthracycline-taxane combination regimen. Third, the NCD lacked standardized pathological assessment criteria between 2012 and 2016, necessitating our use of an alternative pCR definition that may have reduced sensitivity compared to trial-based assessments. Seventh, approximately 30 % of patients had no follow-up data.

Unlike clinical trials showing the efficacy of perioperative drug therapy in early breast cancer [18,19], this study showed a greater impact of AdjC on OS than on RFS. It was because of the characteristics of the database. Recurrence and death are not mandatory entries for obtaining a Board Certified Surgeon by the Japanese Surgical Society. Death is unlikely to be omitted because of the important information that must be entered into the medical record. The omission of recurrence input is a measurement bias that could have been occurred equally in both groups. However, it may have caused fewer events and made it harder to show a RFS difference. In an article which examined the relationship between the effects on time-to-disease progression and OS in metastatic breast cancer, 13 % of the studies resulted in hazard ratios representing a minimal effect on progression with a more pronounced effect on survival, the same as the current study [20].

The results of this study cannot be immediately applied to the current standard treatment for operable TNBC due to limitations. During 2012 and 2016, we couldn't use current standard chemotherapy such as dose-dense regimen [21,22], adjuvant capecitabine for non-pCR [23], and perioperative pembrolizumab [18,19]. However, this study included patients who would not be included in a randomized controlled trial: those with older age, those with multiple comorbidities, those with histological special type, and those from various hospitals all over Japan. So, AdjC might be considered in those who are not candidates for the intensive chemotherapy. In the future, we need the personalized medicine which can identify the patients who are resistant to NAC and who should have surgery first.

## 5 Conclusion

The worse OS and RFS in operable TNBC patients with NAC might be due to unadjusted confounders and low pCR rate. This result has a limited impact on clinical practice, given the current availability of immune checkpoint inhibitors for perioperative chemotherapy. However, it might contribute to the selection of treatment strategies for those who are not candidates for pembrolizumab, such as those with autoimmune disease, cT1cN0, or elderly patients.

It is unclear whether the resection of the primary tumor first has any biological advantage, such as not causing chemotherapy resistance. We need to find biomarkers to identify operable TNBC patients who do not respond to NAC and have poor prognosis.

## CRediT authorship contribution statement

**Tomoe Taji:** Writing – review & editing, Writing – original draft, Project administration, Methodology, Funding acquisition, Conceptualization. **Hiraku Kumamaru:** Writing – review & editing, Writing – original draft, Project administration, Methodology, Investigation, Formal analysis, Data curation, Conceptualization. **Yuki Kataoka:** Writing – review & editing, Writing – original draft, Project administration, Methodology, Investigation, Funding acquisition, Conceptualization. **Kotaro Iijima:** Writing – review & editing, Project administration, Methodology, Conceptualization. **Hirofumi Suwa:** Writing – review & editing, Supervision, Project administration, Methodology, Funding acquisition, Conceptualization. **Hiroshi Ishiguro:** Writing – review & editing, Supervision, Project administration, Methodology, Funding acquisition, Conceptualization. **Naruto Taira:** Writing – review & editing, Supervision. **Takanori Ishida:** Writing – review & editing, Supervision. **Shigehira Saji:** Writing – review & editing, Supervision, Project administration.

## Funding

This study was funded by the 10.13039/100015638Japanese Breast Cancer Society.

## Declaration of competing interest

TT reports a research grant from Astra Zeneca. HK reports receiving consultation fee from EPS Corporation, speaker fee from Chugai Pharmaceutical Co., Ltd, and a research grant on an unrelated subject from Amgen KK and Pfizer KK. HK is affiliated with the Department of Healthcare Quality Assessment at the University of Tokyo, a social collaboration department supported by the National Clinical Database, Johnson & Johnson K.K., Nipro corporation, and Intuitive Surgical Sàrl. HI reports lecture fees from Chugai, Kyowa Kirin, MSD, Eisai, Daiichi Sankyo, Eli Lilly, Astra Zeneca, Taiho, advisory role for Mitsubishi Tanabe Pharma Corporation, Asahi Kasei Pharma and research grants from Eisai, Chugai, Takeda, MSD, Astra Zeneca, Daiichi Sankyo, Nipro corporation, Ono. NT reports a board member of JBCS. SS reports lecture fees from Chugai, Kyowa Kirin, MSD, Novartis, Eisai, Takeda, Daiichi Sankyo, Eli Lilly, Astra Zeneca, Pfizer, Taiho, Ono, Nipponkayaku, Gilead, Exact Sciences, advisory role for Chugai/Roche, Astra Zeneca, Eli Lilly, Pfizer, Kyowa Kirin, Daiichi Sankyo, MSD, research grants from Taiho, Eisai, Chugai, Takeda, MSD, Astra Zeneca, Daiichi Sankyo, Gilead, Eli Lilly, Sanofi and Executive board member of JBCRG, JBCS, JSMO and BIG.

## References

[1] Sørlie T., Perou C.M., Tibshirani R. (2001). Gene expression patterns of breast carcinomas distinguish tumor subclasses with clinical implications. Proc Natl Acad Sci U S A.

[2] Curigliano G., Burstein H.J., Gnant M. (2023).

[3] Asselain B., Barlow W., Bartlett J. (2018). Long-term outcomes for neoadjuvant versus adjuvant chemotherapy in early breast cancer: meta-analysis of individual patient data from ten randomised trials. Lancet Oncol.

[4] Xia L.Y., Hu Q.L., Zhang J., Xu W.Y., Li X.S. (2020). Survival outcomes of neoadjuvant versus adjuvant chemotherapy in triple-negative breast cancer: a meta-analysis of 36,480 cases. World J Surg Oncol.

[5] Fisher C.S., Ma C.X., Gillanders W.E. (2012). Neoadjuvant chemotherapy is associated with improved survival compared with adjuvant chemotherapy in patients with triple-negative breast cancer only after complete pathologic response. Ann Surg Oncol.

[6] Kennedy C.R., Gao F., Margenthaler J.A. (2010). Neoadjuvant versus adjuvant chemotherapy for triple negative breast cancer. J Surg Res.

[7] Bagegni N.A., Tao Y., Ademuyiwa F.O. (2019). Clinical outcomes with neoadjuvant versus adjuvant chemotherapy for triple negative breast cancer: a report from the National Cancer Database. PLoS One.

[8] Clifton K., Gutierrez-Barrera A., Ma J. (2018). Adjuvant versus neoadjuvant chemotherapy in triple-negative breast cancer patients with BRCA mutations. Breast Cancer Res Treat.

[9] Philipovskiy A., Corral J., Dwivedi K.A., Heydarian R., Gaur S. (2019). Efficacy of neoadjuvant versus adjuvant chemotherapy in hispanic/latino (H/L) women with local or locally advanced triple-negative breast cancer (TNBC). In Vivo (Brooklyn).

[10] Kubo M., Kumamaru H., Isozumi U. (2020). Annual report of the Japanese Breast Cancer Society registry for 2016. Breast Cancer.

[11] Japanese Breast Cancer Society (2012).

[12] Lakhani S.R., Ellis I.O., Schnitt S., Tan PH van de V.M. (2012).

[13] Aihara T., Toyama T., Takahashi M. (2016). The Japanese Breast Cancer Society clinical practice guideline for systemic treatment of breast cancer, 2015 edition. Breast Cancer.

[14] Curigliano G, Burstein HJ, Winer EP, et al.De-escalating and escalating treatments for early-stage breast cancer: the st. Gallen International Expert Consensus Conference on the primary therapy of early breast cancer. 10.1093/annonc/mdy537 PMC6637369 30624592

[15] Kaufman C., Sarantou T., Donovan C., Kamali Polat A., Thomas P., Mack B. (2024). ASO author reflections: increasing time of diagnosis to breast cancer surgery. Ann Surg Oncol.

[16] Hanna T.P., King W.D., Thibodeau S. (2020). Mortality due to cancer treatment delay: systematic review and meta-analysis. BMJ.

[17] Mateo A.M., Mazor A.M., Obeid E. (2020). Time to surgery and the impact of delay in the non-neoadjuvant setting on triple-negative breast cancers and other phenotypes. Ann Surg Oncol.

[18] Schmid P., Cortes J., Dent R. (2022). Event-free survival with pembrolizumab in early triple-negative breast cancer. N Engl J Med.

[19] Schmid P., Cortes J., Pusztai L. (2020). Pembrolizumab for early triple-negative breast cancer. N Engl J Med.

[20] Sherrill B., Amonkar M., Wu Y. (2008). Relationship between effects on time-to-disease progression and overall survival in studies of metastatic breast cancer. Br J Cancer.

[21] Mastro L., Poggio F., Blondeaux E. (2022). Fluorouracil and dose-dense adjuvant chemotherapy in patients with early-stage breast cancer (GIM2): end-of-study results from a randomised, phase 3 trial. Lancet Oncol.

[22] Cameron D., Morden J.P., Canney P. (2017). TACT2 Investigators. Accelerated versus standard epirubicin followed by cyclophosphamide, methotrexate, and fluorouracil or capecitabine as adjuvant therapy for breast cancer in the randomised UK TACT2 trial (CRUK/05/19): a multicentre, phase 3, open-label, randomised, controlled trial. Lancet Oncol.

[23] Masuda N., Lee S.J., Ohtani S. (2017). Adjuvant capecitabine for breast cancer after preoperative chemotherapy. N Engl J Med.

