# Supplementary material for: Comparison of neoadjuvant and adjuvant chemotherapy for operable triple-negative breast cancer before the era of immune checkpoint inhibitors: A retrospective study from the Japanese National Clinical Database-Breast Cancer Registry
Source: Breast. 2025 Mar 25;81:104460. doi: 10.1016/j.breast.2025.104460 (PMC11992521; doi:10.1016/j.breast.2025.104460)
Supplement: Multimedia component 1 [file mmc1.docx]

# Figure S1. DAG


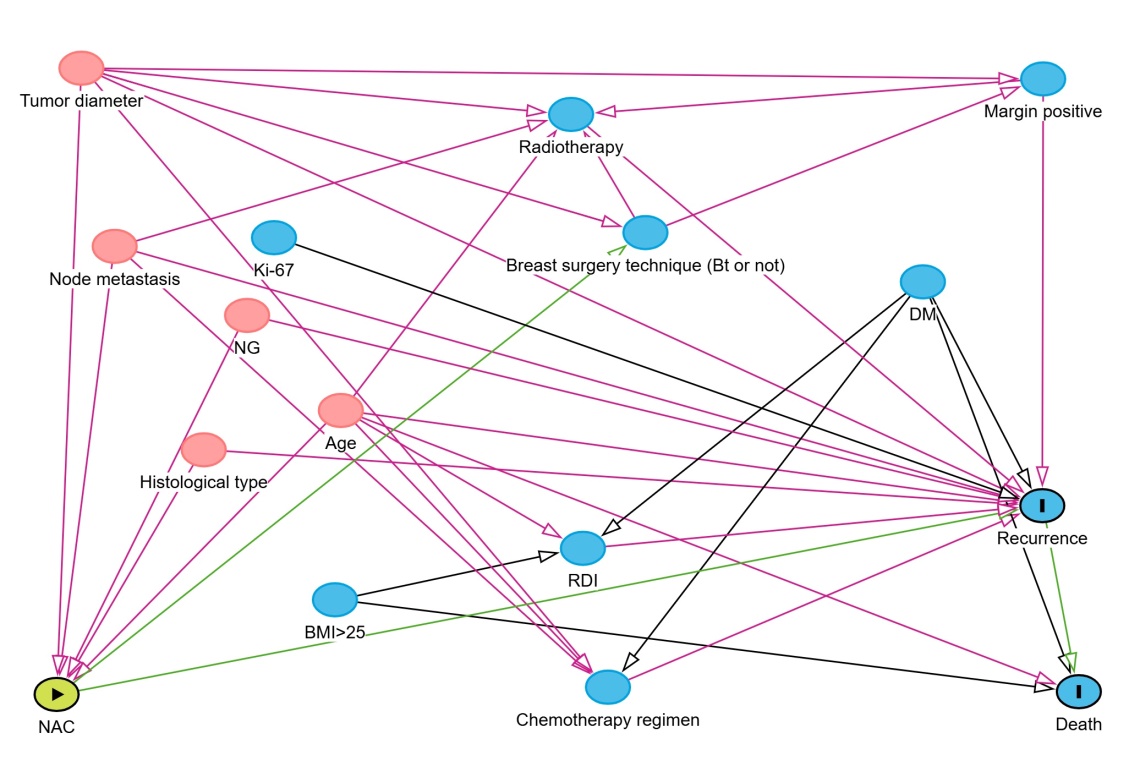


# Figure S2. Yearly trends in the percentage of NAC

|  | 2012 | 2013 | 2014 | 2015 | 2016 | Total |
| --- | --- | --- | --- | --- | --- | --- |
| AdjC | 1898 | 1816 | 1836 | 1808 | 1642 | 9000 |
| NAC | 1071 | 1145 | 1162 | 1116 | 1026 | 5520 |
| Total | 2969 | 2961 | 2998 | 2924 | 2668 | 14520 |

# Figure S3. Kaplan-Meier curves comparing RFS and OS between NAC TNBC patients with p-CR and non-pCR

Pathological complete response is defined as 0 cm or unknown diameter of invasive tumor and 0 lymph node metastasis. The pCR rate was 26.9%.


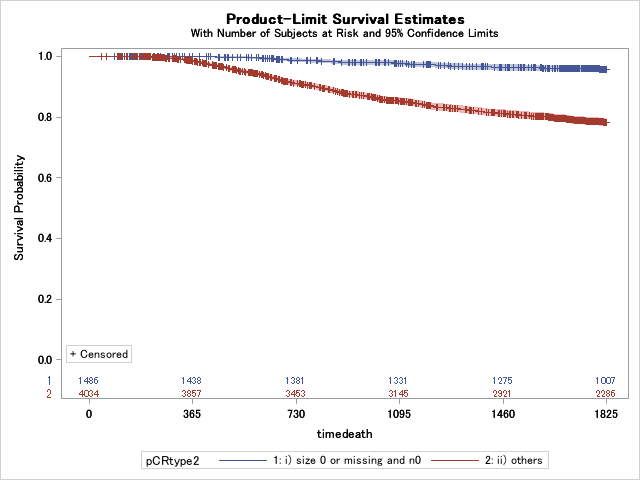


pCR

Non-pCR

**Overall survival**

HR 0.18 (95% CI: 0.14-0.23), p<.0001


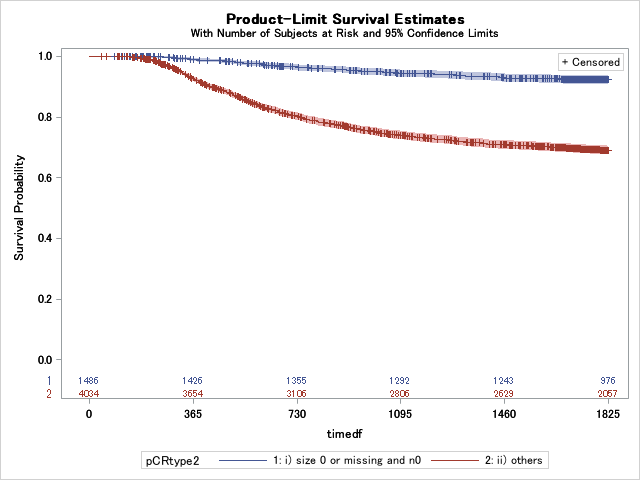


pCR

Non-pCR

HR 0.21 (95% CI: 0.17-0.26), p<.0001

**RFS (recurrence-free survival)**

# Figure S4. Kaplan-Meier curves comparing OS between stage II and III TNBC patients with NAC and those with AdjC.


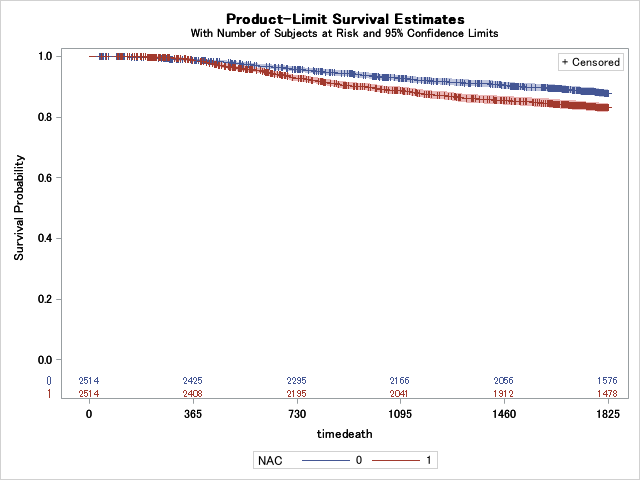


**Overall survival**

NAC

AdjC
