# Supplementary material for: Comparison of neoadjuvant and adjuvant chemotherapy for operable triple-negative breast cancer before the era of immune checkpoint inhibitors: A retrospective study from the Japanese National Clinical Database-Breast Cancer Registry
Source: Breast. 2025 Mar 25;81:104460. doi: 10.1016/j.breast.2025.104460 (PMC11992521; doi:10.1016/j.breast.2025.104460)
Supplement: Multimedia component 2 [file mmc2.docx]

# Table S1. REDORD checklist

See the attached document.

# Table S2. Chemotherapy regimen other than anthracycline and taxane

| Chemotherapy regimen | AdjC (n=1011) | NAC (n=93) |
| --- | --- | --- |
| CMF | 167 | 12 |
| Tegafur uracil | 536 | 4 |
| Tegafur gimeracil oteracil | 106 | 15 |
| Capecitabine | 86 | 13 |
| Carboplatin | 0 | 0 |
| Vinorelbine | 4 | 0 |
| Gemcitabine | 6 | 6 |
| Eribulin | 2 | 1 |
| Others | 107 | 45 |

# Table S3. Time from the start of chemotherapy to surgery as a surrogate for RDI

|  | N | (%) |
| --- | --- | --- |
| Anthracycline and taxane  <126 days  126-210 days  210 days<  Inappropriate | 3609  126  2807  661  15 | 3.5  77.8  18.3  0.4 |
| Anthracycline only or taxane only  <84 days  84-105 days  106 days<  Inappropriate | 901  131  254  510  6 | 14.5  28.2  56.6  0.7 |
